# Datasets of socio-economic, demographic, entrepreneurship, and financial inclusion indicators of selected sites in Ethiopia: Addis Ababa, Dire Dawa, Shirka zone

**DOI:** 10.1016/j.dib.2020.106665

**Published:** 2020-12-15

**Authors:** Abel Tewolde Mehari, Degife Ketema Alemu, Senayit Seyoum Yilma

**Affiliations:** Arsi University, Asella, Ethiopia

**Keywords:** SDGs, Financial inclusion, Entrepreneurship, Unemployment, Ethiopia

## Abstract

Ethiopia is known for having a large portion of its population living under national and international poverty lines. Exclusively the poverty is aggravated being accompanied by a high youth unemployment rate and severe inequality. Thus, these datasets are collected to develop the poverty and unemployment profile of the country with an emphasis on eastern and central regions. Principally the data targeted Addis Ababa: the capital city; Dire Dawa city council- eastern province of Ethiopia and Arsi Zone. The datasets contain demographic variables, household details, education, health & nutrition, employment, non-wage income, death profiles, housing detail, asset ownership, household infrastructure, water & sanitation, household monthly expenditure, saving trends, and social engagement. Besides, the dataset encompasses youth-specific core variables such as finance, unemployment, and entrepreneurship variables. In collecting these datasets, enumerators who have experience in digital data collection were involved. Those enumerators equipped with the digital device were provided two days of digital data collection training, involved in a pilot survey, and finally engaged in the actual data collection activity.

## Specifications Table

**Table:** 

Subject	Social Sciences
Specific subject area	Development
Type of data	Tabular or Matrix form
How data were acquired	Survey Questionnaire The questionnaires are available and accessible at: https://dataverse.harvard.edu/dataset.xhtml?persistentId=doi:10.7910/DVN/W67ZBO#datasetForm:tabView:termsTab https://dataverse.harvard.edu/dataset.xhtml?persistentId=doi:10.7910/DVN/MKCHLN#datasetForm:tabView:termsTab
Data format	Survey Data: Raw
Parameters for data collection	The survey is a population survey in which all target elements in stated administrative regions are accounted for in the dataset. It is a pilot survey conducted to guide and promote the culture of evidence-based decision making among policymakers at grass root administration level. The initiative is termed as Community Based Monitoring Systems (CBMS). For detail refer to the following link: https://www.pep-net.org/sites/pep-net.org/files/typo3doc/pdf/promotionnal_material/CBMS/CBMS_Brochure_12_Pages_Ver.pdf
Description of data collection	The datasets were collected with the help of digital gadgets. Thus, it was easy and successful to gather data from a total household of 5363(two phases average count) or a total of 19746(two phases average count) individuals in eastern and central parts of Ethiopia. The survey was done in two phases. Phase I was implemented on May 10-20, 2014, and phase two was conducted on October 10-20, 2017.
Data source location	Institution: Arsi University-corresponding author: Abel Tewolde Mehari. E-mail:abelmgt2000@gmail.com City/Town/Region: Asella /Oromia Country: Ethiopia Latitude and longitude for collected samples/data: Wereda 10(Addis Ketema sub-city) is located at latitude 9^o^ 02’ 49.03” North and Longitude 38^o^ 42’ 52.98” East. Shirka wereda(Gobessa and Mitana Gado) is located at latitude 7^o^ 36’ 51.43” North and longitude 39^o^ 29’ 34.62” East. The two sites of Dire Dawa are located at latitude 9^o^ 36’ 53.17” North(Melka Jebdu/Kebele01) and Longitude 41^o^ 47’ 07.88” East(Gedenser). Primary data sources: Directly collected from target respondents
Data accessibility	Data are hosted on a public repository Repository name: Harvard data verse Data identification number: https://doi.org/10.7910/DVN/W67ZBO. and Direct URL to the data: https://dataverse.harvard.edu/dataset.xhtml?persistentId=doi:10.7910/DVN/W67ZBO#datasetForm:tabView:termsTab Data identification number: https://doi.org/10.7910/DVN/MKCHLN and Direct URL to the data: https://dataverse.harvard.edu/dataset.xhtml?persistentId=doi:10.7910/DVN/MKCHLN#datasetForm:tabView:termsTab
Related research article	Mehari, A.T., Belay, C.F. Challenges and prospects of entrepreneurship development and job creation for youth unemployed: evidence from Addis Ababa and Dire Dawa city administrations, Ethiopia. *J Innov Entrep* 6, 11 (2017). https://doi.org/10.1186/s13731-017-0070-3

## Value of the Data

• Particularly phase II datasets are related to Sustainable Development Goals (SDGs) and it assists stakeholders (including government) to be aware of the current status of the country in relation to the goals. Thus, stakeholders’ can utilize these resources to frame strategies for better accomplishment of the SDGs in the years to come.
• The data can be used by policymakers to identify the reasons behind youth financial exclusion. Thereby it helps policymakers to assess the need for amendment of existing financial policies.
• The data can help to identify and act on the factors significantly attributing to entrepreneurial development and employment creation (This is unique to Dire Dawa and Wereda10).
• Researchers and academicians will benefit from the broad spectrum of variables in the dataset. They can easily process the data and generate relevant journal articles related to poverty, inequality, finance, and SDGs.

## 1 Data Description

The two datasets are available labelled as phase I and Phase II. These datasets are available along with relevant data dictionaries (codebooks) and data collection instruments. Here below are descriptions of the datasets:

***Phase I:*** Consists of all data sets, questionnaires, training manuals, and data dictionaries in which CBMS Ethiopia used to collect relevant socio-economic and demographic variables of selected wereda^1^ in Addis Ababa and Dire Dawa. It has also a household-level dataset encompassing the following variables: Family size, Health, Employment, Source of income, Housing detail, Asset ownership, Household infrastructure, Water and sanitation, Household consumption, Saving, Social engagement. Individual Dataset consists of demographic variables, death, financial services, entrepreneurship, and unemployment. Besides, it has a death dataset with variables such as gender & age of the deceased person and medical reason for the reported death.
Finally, this set (Phase I) encompasses merged individual and household data. This is an intentionally merged dataset of individual and household-level data to enable researchers and users to have a list of broad variables and flexibility of analysis. The researcher might merge those separate data with mainid as a key variable and generate this data by themselves. But we prefer to help and avoid duplication of efforts.
***Phase II:*** Like phase I, almost all files of this set are Data sets, questionnaires, training manuals, and data dictionaries. The unique aspect of this set is all profiles are from selected wereda of Addis Ababa and Shirka wereda and little modifications^2^ have been made on the questionnaires and corresponding variables in the dataset and the data dictionaries.

## 2 Experimental Design, Materials and Methods

This section vividly elaborates the due process of questionnaire design, data collection process, data types, and technical validation implemented to secure the quality of collected datasets.

### 2.1 Questionnaire design

The questionnaires were designed in such a way that they encompass questions about demographics, health, education, consumption, and income. Initially, two actual set of questionnaires were developed, but they were later merged as a single questionnaire at the onset of actual data collection on the digital format. Note that the nature of the paper questionnaire is quite different from the digital version (possible to be delivered this based on request). Before converting them to digital format, pilot testing was conducted on the two sets of paper questionnaires using 50 observations, and consequent revision is made.

Then a long time and unreserved efforts have been exerted to change the paper questionnaire to a digital version. The digital version has the capability of skipping modes of questions that are not relevant based on the reply of preceding questions. Moreover, it helps the enumerator to make sure that all questions are appropriately and completely responded.

The questions in the digital format are designed to have logical linkage among themselves and some of them allow a single reply (circle radio button) and the rest of them allow multiple replies (tick box). These digital reply interfaces (circle radio button &tick box) are designed and incorporated into the questionnaires based on the general android convention. The general android convention dictates circle radio button shall be set for the questions where the respondent is supposed to reply for a single option (answer). Whereas a square tick box shall be set for the questions one is supposed to provide multiple answers. Radio buttons are very useful when there is a need to select from among mutually exclusive items. Checkboxes are different from the radio button where the respondent is allowed to select more than one reply. Text input as a response format needs to consider the size of the box. The usage of scrolling around the text box if possible should be avoided. This type of input control is very useful in an open-ended questions [1], [2], [3], [4].

The windows version and android version digital questionnaires are available on the webserver of De La Sale University, it can only be accessed upon request and by contacting the authors. The digital questionnaire in addition to having circle and square radio buttons, also has ‘pulled down’ reply options.

### 2.2 Data collection framework and data types

**Fig. 1:**
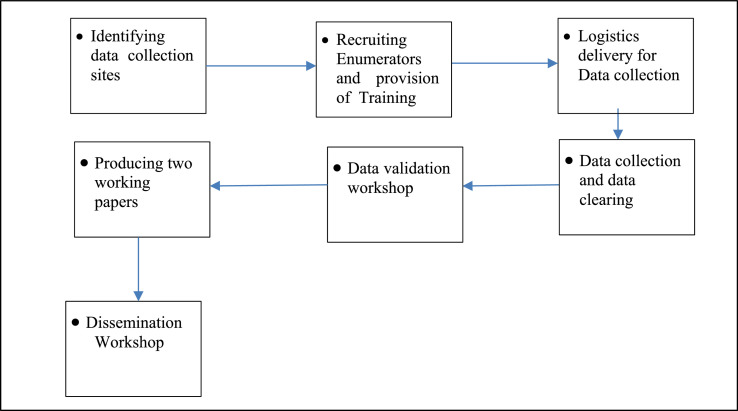
Framework of the data collection.

The framework in Fig.1 shows the chain of activities implemented to secure the valuable data shared in this article. The authors made formal contact with local administrators to identify the boundary of the localities and create awareness about what is supposed to be done and then dates for subsequent tasks have been fixed.

Then 12 enumerators were recruited and relevant data collection training has been conducted. For the sake of convenience and in order to develop a sense of ownership, two skilled enumerators were involved from the locality. The data collection implemented a conventional on-field supervisory control and post data collection validation workshop to secure the quality of the data. Using these datasets, so far two relevant papers have been produced and presented to a panel of experts. However, still a major section of the datasets is untouched and could be an asset for future investigation.

Though not an exhaustive list, Table 1 summarizes and presents the features of the datasets available for sharing with the academic and research community:

**Table 1:** Plain description of the dataset acquired through phase I and phase II of the data collection.

Category	Related questions
Demographic	Age,Gender,Marital status,Religion
Education	Maximum educational achievement
Health	Types of Disability, Access to a health facility, addiction to cigarettes and K'hat
Sanitation	Access to different types of toilet facility and waste disposal schemes
Income	Income in ETB disaggregated agricultural, manufacturing, service, transfers from abroad and government
Consumption	The quantity consumed and price of various types of commodities including recurrent food items, clothing, and other monthly and annual expenditure items.
Social capital	Involvement in communal institutions like edir and equb.

### 2.3 Modes of surveying

The survey used the conventional enumerators' based procedure with the help of digital gadgets to gather data from the total household of 5363(two phases average count) or a total of 19,746(two phases average count) individuals in eastern and central parts of Ethiopia. The survey was done in two phases.

The enumerator's manual (shared and available on the repository) was handover to each enumerator in addition to the intensive training provided before they depart for the actual data collection. The manual informs the enumerators that they have to enumerate the data from those whose age is above 18. Specifically for household-level questions, it is advised to be collected from the head of the household. If the household is not available at the time of the visit, caution is made to leave the premises and plan for a revisit.

The second questionnaire set (Youth related individual level questionnaire) was merged with the household level questionnaire. For instance, if the demographic section of the household questionnaire shows there is a household member whose age ranges from 15 to 29 years old, the gadget will automatically cascade the youth questionnaire for completion else it will keep as hidden.

Within the youth questionnaire sections, there are two different subsections: Financial Inclusion and Entrepreneurship related subsections. In this questionnaire set, there are questions in which the gadget automatically cascades out and in contingent on antecedent replies.

**Fig. 2:**
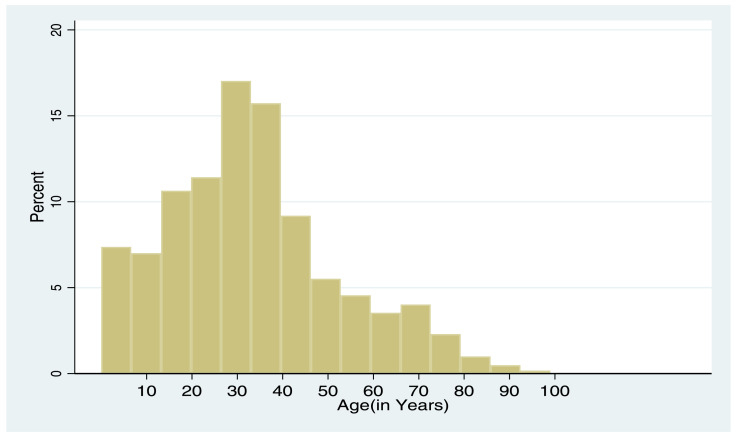
Histogram showing age distribution of respondents in phase II (2017) of data collection.

The following histogram and pie-chart represent the age and gender distribution of individuals enumerated in those data collection sites by phase I and phase II, respectively:

Fig. 2 depicts majority of the respondents are with the age range of 30–40 years old. As it is depicted by the lower bin of the histogram, the maximum age is around 100 and the minimum age is below 10.

**Fig. 3:**
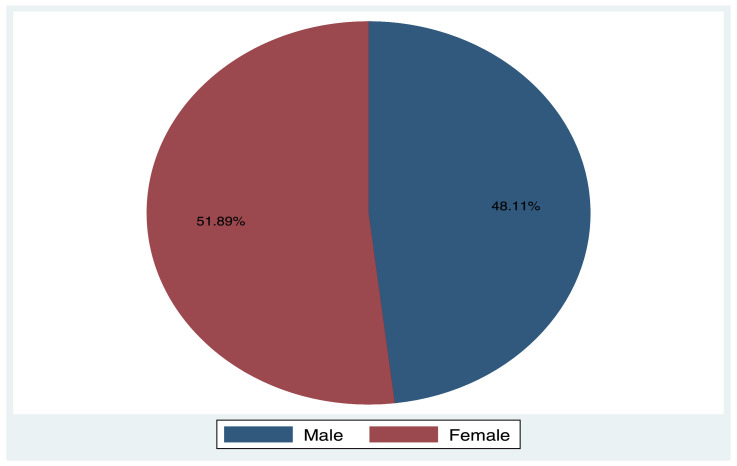
Gender of individuals assessed in the phase II (2017) of data collection.

### 2.4 Data records

Originally the raw data have been uploaded directly from the field to the server located in the Philippines which is managed by Community Based Monitoring System (CBMS) International Network. Currently, these data are not accessible from the stated server. However, these datasets (The Phase I and Phase II datasets) are available at Harvard Dataverse [5,6], from where they can be downloaded as a zip folder.

The datasets are downloadable in a zip format and extractable using a freely available zip extractor.

In this repository ‘***.dta’*** format and ‘***.csv’*** format of phase I and phase II datasets are accessible. Moreover, ancillary documentation like data dictionaries, training manuals, and questionnaires are incorporated along with the datasets.

Those ‘***.csv’*** format datasets have been arranged in matrix form where the row indicates observations (household and individual) and columns indicate reply to each question. The replies to the questions are either in a categorical or continuous format. Some categorical replies are binary and the rest are multiple responses. The string form of responses is also available for certain categorical responses. Especially for the questions where multiple responses are allowed string replies of True/False is applied. The continuous variable replies are related to income, consumption, and age.

The original data records hold personal identification like latitude, longitude, and altitude. However taking into consideration of ethical issues and possible repercussions on the observations (participants), we intentionally eliminated them from the datasets.

### 2.5 Technical validation

Maximum efforts have been made to minimize possible errors committed in the field enumeration process. The following technical arrangements were made to reduce the chance of the occurrence of errors.

A Before the actual field enumeration starts, a long time has been spent on designing the questionnaires to encompass skipping mechanisms and logical linkage among the questions. Those skipping and logical linkage procedures help in minimizing the efforts and time spent in the enumeration process. This is considered as one of the mechanisms of pre-collection error avoidance.
B One of the common challenges in conventional pen-paper data enumeration is incomplete and misfed fields (questions). For instance, logically the answer for age should not be negative and should not be in thousands. With the application of vital technical procedures and using a software algorithm, the likelihood of occurrence of this type of error is highly reduced. For example, if erroneously the enumerator feeds negative figures or thousands of digit, the gadget or the algorithm automatically blocks the entry and requests him/her to revise by alarming the error section in red color. Likewise, the algorithm of the digital version of the data collection instrument used is restrictive by nature. Thus, the enumerator can't pass from one page to another page of the questionnaire unless the red error message on the spotted page is resolved.
C Another concern was the post-data collection process of data clearing and data management. The core objective of the data collection is to generate gender, location, age and disability disaggregated outputs. The data are collected and documented in a separate set at household and individual levels. Thus, merging, appending, and collapsing might be required to do disaggregated and aggregated analyses. On the working papers which have been produced for Partnership for Economic Policy (PEP) publication, STATA syntax 1: M, M:1, 1:1, and M:M data merging option were used wherever they are necessary. Likewise, it is technically advisable to append and merge data if a broad and strong analysis is intended. Those technical procedures (appending and merging) are possible using mainid as a key variable.
D The commitment we took to collect data related to malnutrition justifies the quality of the dataset. With the advice we get from local health officers we incorporated Mid Upper Arm Circumference (MUAC) measurement kit. The MUAC kit with red, yellow, and green colors shows Severely Malnourished, Mild Malnourished, and Not malnourished, respectively [7], [8].

**Fig. 4:**
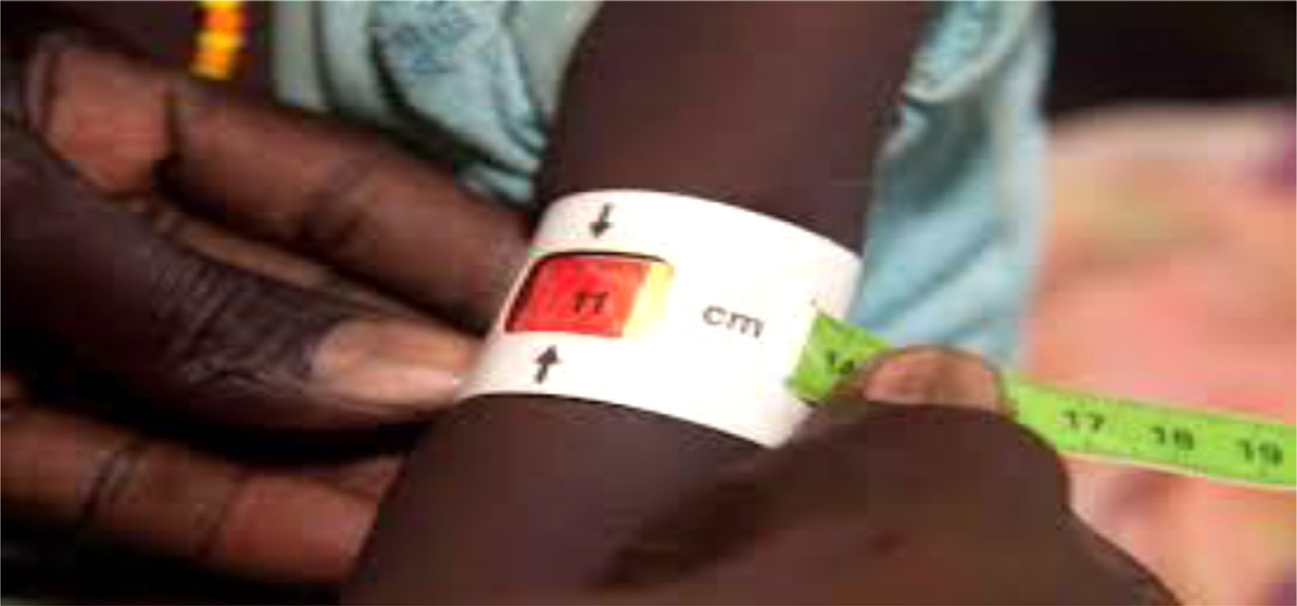
Mid Upper Arm Circumference (MUAC).

Fig. 4 demonstrates the way the kit is used to reveal child malnutrition status. In this figure, the child is severely malnourished which is signaled by the red label. Note that Fig. 4 is not the actual picture we capture from the data collection sites. For the sake of not violating our respondent's privacy, we are not able to capture and disclose a similar picture.

## Ethics Statement

In a scenario where datasets are obtained by not involving human experiments, non- interventional investigation, the anonymity of participants is kept confidential, ethical approval exemption is possible. Thus, based on Ethiopian National Ethics Guideline section 7.2 automatic exemption without prior documentation is applicable for these datasets [9]. Moreover, note that prior informed consent has been obtained from each respondent while enumerators were on the field process.

## CRediT Author Statement

**Abel Tewolde Mehari:** Funding acquisition, Project administration, Visualization, Methodology, Investigation, Data Curation, Writing - Original Draft.

**Degife Ketema Alemu:** Project administration, Validation, Formal analysis, Investigation, Resources, Writing - Original Draft.

**Senayit Seyoum Yilma:** Investigation, Writing - Original Draft.

## Declaration of Competing Interest

The authors declare that they have no known competing for financial interests or personal relationships which have, or could be perceived to have, influenced the work reported in this article.

## Footnotes

1 Next to kebele , it is the second lowest level administrative council in Ethiopia.

2 *The questionnaires, codebook, and manuals of phase II are modified compared to Phase I. Specially the Household Profile Questionnaire(HPQ) is design based on set of Sustainable Development Goals(SDGs) formulated by United Nations.*

## References

[1] Checkbox survey Inc., Checkbox 6 Question Items Guide. http://checkbox.resources.s3.amazonaws.com/documentation/v6/Question%20Items%20Guide/Checkbox-6-Question-Items-Guide.pdf, 2013 (Accessed 20 August 2020).

[2] EU survey, EUSurvey 1.4.1 – Quiz Guide. https://circabc.europa.eu/sd/a/400e1268-1329-413b-b873-b42e41369a07/EUSurvey_Quiz_Guide.pdf, 2019 (Accessed 20 August 2020).

[3] Qualtrics,Multiple Choice Questions. https://www.qualtrics.com/support/survey-platform/survey-module/editing-questions/question-types-guide/standard-content/multiple-choice/, 2019 (Accessed 20 August 2020).

[4] Z. Saaya, A. Devaraju, N. Mustafa, C. Leong, The Implementation of Quationnaires Design Principles via Online., Faculty of Information and Communication Technology,Universiti Teknikal Malaysia Melaka, n.d.

[5] A.T. Mehari, D.K Alemu, S.S. Yilma, Dataset Phase I,Harvard Dataverse,V3.3, 2020. 10.7910/DVN/W67ZBO.

[6] A.T. Mehari, D.K Alemu, S.S. Yilma, Dataset Phase II Harvard Dataverse, V2.2, 2020. 10.7910/DVN/MKCHLN.

[7] Walter T., Sibson V. (2012). Mid Upper Arm Circumference and Weight-for-Height Z-Score as Indicators of Severe Acute Malnutrition: a Consultation of Operational Agencies and Academic Specialists to Understand the Evidence, Identify Knowledge Gaps and to Inform Operational Guidance.

[8] Tang A., Dong K., Deitchler M., Chung M., Maalouf-Manasseh Z., Tumilowicz A., Wanke C. (2013). Use of Cutoffs for Mid-Upper Arm Circumference (MUAC) as an Indicator or Predictor of Nutritional and Health Related Outcomes in Adolescents and Adults: A Systematic Review.

[9] Ministry of Science and Technology (2014). National Research Ethics Review Guideline.

